# The implications of spatially variable pre‐emergence herbicide efficacy for weed management

**DOI:** 10.1002/ps.4784

**Published:** 2017-12-14

**Authors:** Helen Metcalfe, Alice E Milne, Richard Hull, Alistair J Murdoch, Jonathan Storkey

**Affiliations:** ^1^ Rothamsted Research Harpenden UK; ^2^ School of Agriculture, Policy and Development University of Reading Reading UK

**Keywords:** Alopecurus myosuroides Huds. (black‐grass), pre‐emergence herbicides, soil organic matter, sublethal effects, pendimethalin, flufenacet

## Abstract

**BACKGROUND:**

The efficacy of pre‐emergence herbicides within fields is spatially variable as a consequence of soil heterogeneity. We quantified the effect of soil organic matter on the efficacy of two pre‐emergence herbicides, flufenacet and pendimethalin, against Alopecurus myosuroides and investigated the implications of variation in organic matter for weed management using a crop–weed competition model.

**RESULTS:**

Soil organic matter played a critical role in determining the level of control achieved. The high organic matter soil had more surviving weeds with higher biomass than the low organic matter soil. In the absence of competition, surviving plants recovered to produce the same amount of seed as if no herbicide had been applied. The competition model predicted that weeds surviving pre‐emergence herbicides could compensate for sublethal effects even when competing with the crop. The ED50 (median effective dose) was higher for weed seed production than seedling mortality or biomass. This difference was greatest on high organic matter soil.

**CONCLUSION:**

These results show that the application rate of herbicides should be adjusted to account for within‐field variation in soil organic matter. The results from the modelling emphasised the importance of crop competition in limiting the capacity of weeds surviving pre‐emergence herbicides to compensate and replenish the seedbank. © 2017 The Authors. *Pest Management Science* published by John Wiley & Sons Ltd on behalf of Society of Chemical Industry.

## 1 INTRODUCTION

Herbicides are an important component of weed control programmes and soil‐applied herbicides are particularly important for controlling germinating weeds in the context of the rapid evolution of resistance to foliar‐applied herbicides. Generally, these pre‐emergence herbicides are applied uniformly across the field at doses recommended by the manufacturer. These recommended doses are given irrespective of variation in soil properties and for many pre‐emergence herbicides the only advice, with regard to soil, is that they should only be used on soil with organic matter up to 10%. In the United Kingdom (UK), this is seldom problematic, given that the average soil organic matter (0–15 cm) in arable and horticultural land is 3.07%.^1^

*Alopecurus myosuroides* Huds. (black‐grass) is a particularly problematic weed of winter wheat (*Triticum aestivum* L.) in the UK and so its control is of concern. Given increasing problems of evolving resistance to contact herbicides,^2^ and the decreasing number of options available for chemical control, the currently available pre‐emergence herbicides (based largely on flufenacet and pendimethalin) are an increasingly important tool in the arsenal against this pernicious weed. *Alopecurus myosuroides* exhibits patchy distributions within fields, yet its control is often through uniform application of herbicides. As with many species, it is thought that these patchy distributions in arable fields are strongly affected by their environment, in particular, the soil.^3^, ^4^

Soil properties not only affect the life‐cycle of the weeds directly^5^ but they can also have an indirect effect by altering the efficacy of some herbicides.^6^ Organic matter in the soil can lead to adsorption of herbicide.^7^ Different herbicides may be more, or less, adsorbed by organic matter, dependent on their physical and chemical properties,^8^ with flufenacet and pendimethalin both adsorbing highly to the soil (pendimethalin *K*_d_ = 2.23 to 168^9^ and flufenacet *K*_d_ = 0.77 to 4.52^10^). If the amount of soil organic matter varies across the field, there may be parts of the field where these pre‐emergence herbicides are less available to the plant. This, in turn, may lead to differential control across the field and so increases the chance of the establishment of weed patches where herbicide control is reduced.

As pre‐emergence herbicides are applied directly to the soil, it is particularly important to understand how varying soil properties within fields may affect their efficacy. Some studies have considered this and shown that organic matter influences herbicide efficacy. For example, Nordmeyer^8^ showed that a relatively small increase in organic matter from 2.2 to 3.5% can impact the efficacy of chlortoluron against *A. myosuroides*. Blumhorst *et al*.^11^ also demonstrated a strong correlation between soil properties and the herbicidal activity of five different herbicides against *Abutilon theophrasti* Medik. (velvetleaf) and *Setaria viridis* L. (green foxtail). Despite this previous research indicating that varying organic matter in the soil can lead to reduced weed control, little has been done to understand the response of two key pre‐emergence herbicides in the control of *A. myosuroides* to such changes in soil organic matter and the implications for weed management in terms of potential weed seed return.

Given that in a field with spatially heterogeneous organic matter we would expect different levels of control, it is important to consider any sublethal effects on the survivors and their impact through the rest of the growing season. When implementing weed control strategies, the focus is often on diminishing seed return. Modelling is frequently used to investigate the effects of different management practices (e.g. cultivation^12^, ^13^, crop rotations^14^ and patch spraying^15^). Where these models include the use of herbicide, however, they generally model the effect of herbicide simply as a proportional kill of weed seedlings, with the implicit assumption that the survivors remain unaffected^16^. Sublethal effects have been shown to be important in many species; for example, Rotchés‐Ribalta *et al*.^17^ showed that some species show no decrease in biomass at harvest irrespective of the dose of herbicide received and Riemens *et al*.^18^ showed that with increasing dose the number of seeds per gram of fresh weight can decrease. Often when field data are collected on herbicide efficacy there is a marked difference between the levels of control achieved at the seedling stage and the head stage.^19^ It cannot, therefore, be assumed that pre‐emergence efficacy at the seedling stage is equivalent to a proportional decrease in seed return in the absence of subsequent herbicide activity. Variability in pre‐emergence herbicide activity needs to be understood in the context of the effect of soil heterogeneity on the rest of the weed life‐cycle.

Our aim was to quantify the effect of variable soil organic matter on the seed return of *A. myosuroides* following the application of flufenacet or pendimethalin at a range of doses. We examined both the level of control achieved by those two herbicides on soil with different amounts of organic matter as well as sublethal effects of the herbicide, and the ability of plants to recover and produce viable seed in the context of crop competition. To investigate this, we considered three different levels of organic matter, typical of arable fields in the UK, and a range of herbicide dose rates applied to *A. myosuroides* seedlings in pots. We hypothesized that increasing organic matter would lead to decreased efficacy of both herbicides in the control of *A. myosuroides* and that, where there were survivors, sublethal doses would lead to fitness costs causing reduced growth and fecundity (the fitness cost also being determined by soil properties). We used regression analyses to determine whether soil organic matter impacts the shape of the dose–response curves for these two herbicides observed in pots and used the results from the pot experiment to parameterise a simulation model of crop–weed competition and weed seed production in the field. An increased understanding of how local soil conditions affect the efficacy of pre‐emergence herbicides and the implications for *A. myosuroides* population dynamics will increase our ability to properly manage this prolific agricultural weed, particularly in the context of precision weed control and integrated weed management strategies.

## 2 METHODS

### 2.1 Soil

To isolate the effects of soil organic matter from the many covarying soil properties, we created three artificial soils with varying amounts of organic matter, whilst maintaining other soil properties at relatively constant values by mixing sand, loam, and composted bark in different ratios (Table 1). Composted bark is homogeneous in its organic matter content and so allowed fine adjustment of soil organic matter whilst adding minimal additional nutrients. Samples of each soil mixture were tested by the laboratories at SOYL (Newbury, UK) using loss on ignition to establish the amount of organic matter (Table 1). The range of organic matters achieved was typical of UK arable land. We created two soils with organic matter <3% (typical of British arable soil) and one soil representing particularly high levels but still within the 10% level quoted on many herbicide labels. Each soil type was used to fill 180 10‐cm‐diameter pots and 90 25‐cm‐diameter pots, giving a total of 540 10‐cm‐diameter pots and 270 25‐cm‐diameter pots.

**Table 1:** The volumetric composition of the three soil mixtures used, their percentage organic matter (OM) measured by loss on ignition (LOI), and the measured pH of the soils

Soil mixture	Coarse sand (% by volume)	Fine sand (% by volume)	Loam (% by volume)	Composted bark (% by volume)	OM (LOI % w/w)	pH
Low organic matter	35	35	22.5	7.5	1.93	7.16
Medium organic matter	20	20	45	15	2.37	7.00
High organic matter	0	0	75	25	6.15	7.00

### 2.2 Plant material

We used *A. myosuroides* seed from a plot on the Broadbalk long‐term experiment established at Rothamsted Research (Harpenden, UK) in 1843, which had never received any herbicides.^20^ This population has been shown to be free of any evolved herbicide resistance. The seed was collected in 2014 and had been stored in darkness from harvest until the start of this experiment. We germinated *A. myosuroides* seeds in Petri dishes in a Sanyo (Osaka, Japan) MLR‐350 environmental test chamber providing a 17 °C 14‐h day, 11 °C 10‐h night for 7 days. Seeds were germinated in Petri dishes lined with three Whatman No.1 90‐mm‐diameter qualitative filter papers and 5 ml of KNO_3_ (2 g l^‐1^). We transplanted eight germinated seeds (radicle just emerged) into each 10‐cm‐diameter pot on 23 February 2016. We placed the pots in an unheated glasshouse and allowed the plants to grow for 6 weeks prior to assessment. All plants received water as required.

### 2.3 Pre‐emergence herbicides

Flufenacet (Bayer CropScience Ltd, Monheim am Rhein, Germany) and pendimethalin (BASF plc, Ludwigshafen, Germany) are two active ingredients present in pre‐emergence herbicide products and are widely used in UK cereals. They are registered in several countries for the control of most annual grasses and common weeds in cereals, fruit and vegetables. Here we tested their efficacy against *A. myosuroides*. Pendimethalin is a residual dinitroaniline herbicide [Herbicide Resistance Action Committee code (HRAC): K1] and flufenacet is an oxyacetamide herbicide (HRAC: K3). We applied each herbicidal active ingredient (Suspension Concentrate formulation) separately pre‐emergence, (1 day post sowing before any shoots had emerged) using a laboratory track sprayer delivering 222 L ha^‐1^ at 210 kPa through a Teejet (Glendale Heights, IL, USA) 110015VK ceramic nozzle, 50 cm above the soil surface. We applied a full range of doses with rates of 0, 1/64, 1/32, 1/16, 1/8, ¼, ½, 1× and 2× recommended field rates (UK) of 240 and 1200 g a.i. ha^‐1^ for flufenacet and pendimethalin, respectively. For each herbicide, each dose was applied to 10 pots of each soil type. The experimental design was a randomized complete block design with 10 replicates of each treatment combination (each herbicide at each dose on each soil; 540 pots in total).

### 2.4 Herbicide efficacy

Six weeks after the application of the pre‐emergence herbicides, we assessed survival as the proportion of individuals that remained alive in each pot. We assessed the average size of surviving plants by counting the number of tillers present in each pot and dividing that by the number of survivors to give an indication of the growth stage of the plants. We measured biomass on five randomly selected replicates by cutting all plant material at ground level and taking a total dry weight per pot.

### 2.5 Sublethal effects

To consider the sublethal effects of flufenacet and pendimethalin and how these are affected by soil organic matter as the plant matures, we kept five replicates of each soil–herbicide–dose combination. These were not destructively assessed for biomass. Where there were survivors, we selected the median sized plant from each pot, and transplanted it into a 25‐cm‐diameter pot containing the same untreated soil mixture. These plants were grown outside under netting. The same randomization as in the glasshouse was maintained. Plants were watered as required. No additional inputs were added to the soil; however, fungicides and insecticides were applied as required and any emerging seedlings were removed by hand.

We recorded the Julian day of first flowering for each of these plants. Once all plants had flowered, we measured the height of the tallest tiller and the number of seed heads produced (27 July 2016). On 24 August 2016, we cut all biomass at ground level and measured the total dry straw biomass. To assess the effect of soil organic matter on fecundity, we measured the total fresh weight of the seed heads and calculated total dry weight from a subsample of seed. We also measured the viability of the produced seed in a germination assay: following 3 months' storage in darkness at 18 °C and 35% humidity, we assessed the viability of the seed produced by assessing germination in Petri dishes. We set up three Petri dishes for each sample with three Whatman No.1 90‐mm‐diameter qualitative filter papers per dish. We put 50 seeds, representative of the uncleaned sample, and 5 ml of KNO_3_ (2 g l^‐1^, Riedel‐deHaen analytical grade) into each dish and then incubated the dishes in a Sanyo MLR‐350 environmental test chamber delivering a 17 °C 14‐h day, 11 °C 10‐h night for 14 days. Following incubation, we counted the number of seeds that had germinated (visible radicle).

### 2.6 Data analysis

We used the *drc* package in r 21 to find the best model to describe the dose–response relationships investigated in our experiments (ignoring treatment structure). We chose from log‐logistic (three, four and five parameters), Weibull (type 1 and 2, with three and four parameters) and Cedergreen–Ritz–Streibig (four parameters; A, B and C types; in all types the lower limit is fixed to 0) (Table 2), as well as linear, quadratic and cubic models to capture any departures from these typical dose–response functions. Using the chosen best‐fit model for a given data set, we assessed the significance of each treatment. First, we allowed the parameters of the model to depend on herbicide to test the hypothesis that the dose–response curves for flufenacet and pendimethalin were different from one another. We then allowed the parameters of the model to depend on soil type to test the hypothesis that the amount of organic matter in the soil affects the dose–response. If allowing the parameters to depend on both herbicide and soil type significantly improved the model (*P* < 0.05), we then assessed the significance of including an interaction term. For the best‐fitting model, we checked each parameter for its importance by setting it to a common value across treatments and testing if the residual sum of squares was significantly altered. If not, the parameter was fixed and the next parameter assessed. We assessed parameters sequentially, beginning with the asymptotes before considering any gradient and timing parameters.

**Table 2:** The models considered for describing the dose–response relationships

Model	Expression	Parameterisation
Log‐logistic	$f\left(x\right)=c+\frac{d-c}{{\left(1+\exp\left(b\left(\log\left(x\right)-\log\left(\eta \right)\right)\right)\right)}^{\gamma }}$	Five parameters
		Four parameters, *γ* = 1
		Three parameters, *c* = 0
Cedergreen–Ritz–Streibig	$f\left(x\right)=\frac{d+\gamma \exp\left(-1/x^{a}\right)}{1+\exp\left(b\left(\log\left(x\right)-\log\left(\eta \right)\right)\right)}$	A type, *α* = 1
		B type, *α* = 0.5
		C type, *α* = 0.25
Weibull type 1	*f*(*x*) = *c* + (*d* − *c*) exp(− exp(*b*(log(*x*) − log(*η*))))	Four parameters
		Three parameters, *c* = 0
Weibull type 2	*f*(*x*) = *c* + (*d* − *c*)(1 − exp(− exp(*b*(log(*x*) − log(*η*)))))	Four parameters
		Three parameters, *c* = 0

### 2.7 Modelling

In our experiments, the potential for seedlings surviving sublethal effects of pre‐emergence herbicides to mature and produce fresh seed was quantified. However, the experiment did not include competition with the crop to avoid confounding the effects of soil organic matter on competitive ability and the ability to recover from sublethal doses. However, without including competition, the experiment did not fully capture the potential fitness penalty of reduced seedling size and the capacity of weeds to compensate for sublethal herbicide effects when competing with a crop. Given that inter‐specific competition is asymmetric, we would hypothesise that survivors would be disproportionately impacted by competition from the crop and this effect would increase disproportionately with greater herbicide efficacy. To challenge this hypothesis, we combined data from the pot experiment with a simulation model of crop–weed competition, which has been parameterised and validated for *A. myosuroides* in winter wheat.^22^ The model is weather driven and operates on a daily time step. Before the onset of competition at canopy closure, weed growth is modelled as an exponential function of effective day degrees,^23^ after which competition for resources is modelled using functions developed in the INTERCOM model.^24^ Weed seed production is calculated from the allometric relationship with mature weed biomass.

The model was initialised with a weed seedbank density of 5000 seeds m^‐2^, 20% of which are in the upper soil layer, from which all seeds are available for germination; the remaining seeds have a smaller chance of germination according to a linear relationship with soil depth. Wheat density was set to 300 plants m^‐2^ and crop and weed emergence was set to 30 September – typical of agronomic practice for the UK. The number of emerged weed seedlings was calculated using a proportion sampled from a distribution function parameterised from a series of field experiments.^25^ A proportion of these seedlings were killed using the mortality figure given by the best model fit for each treatment combination observed in the pot experiments. The mature biomass and seed production of the surviving weed plants were simulated using ten years of weather data measured at Rothamsted Research (Hertfordshire, UK) between 2006 and 2015 capturing seasonal variability in the competitive balance between the crop and weed.^22^ For each year, the model was run 100 times using a new value for proportional germination sampled from the Weibull probability distribution (skewed towards lower emergence) to introduce stochasticity to do with variability in establishment.

The model was used to run two scenarios for each soil–herbicide–dose combination included in the pot experiments: (i) with crop competition, and (ii) without crop competition. Sublethal effects were included in the simulations by reducing weed seedling biomass and green area at the end of the exponential growth phase by the proportion predicted using the dose–response model that fitted the data from the pot experiments best. The output seed production from the simulation model was than analysed in the same way as the data from the experiments and a dose–response curve fitted to see if the different levels of survival and sublethal effects experienced on different soil and following the application of different herbicides at a range of doses can affect the resulting seed production.

## 3 RESULTS

**Figure 1:**
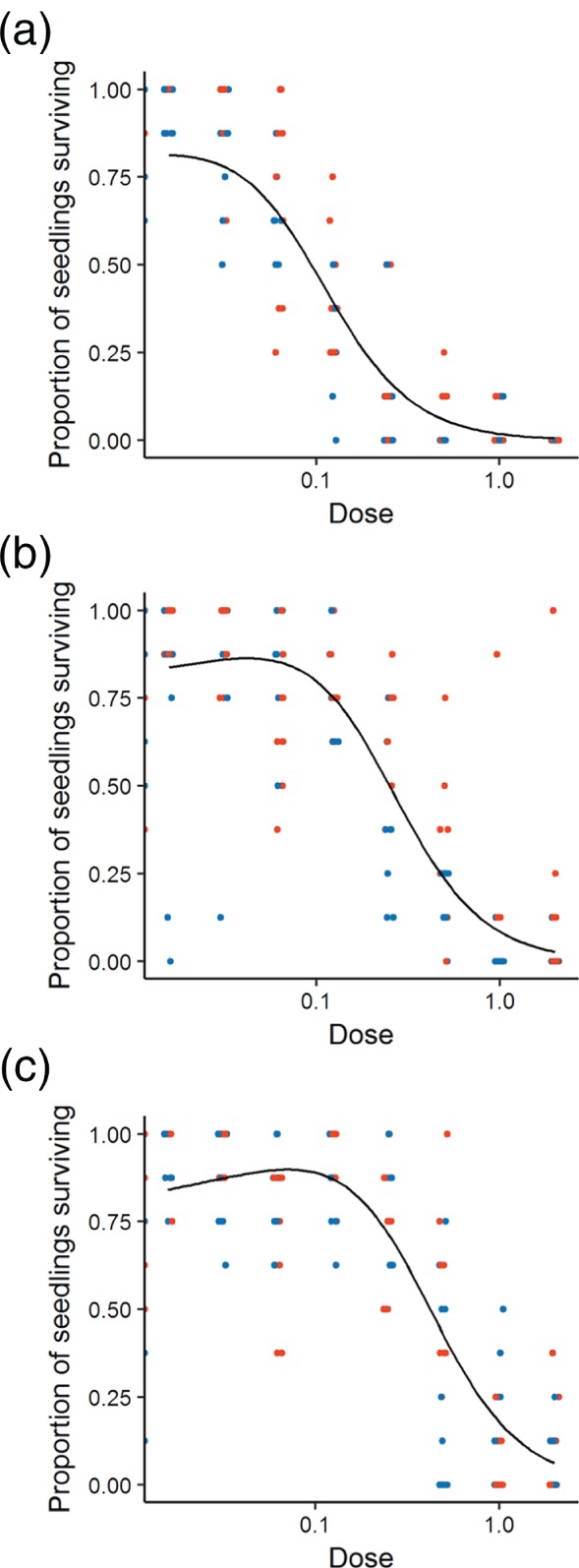
The proportion of seedlings surviving 6 weeks after the application of two pre‐emergence herbicides on soil with varying levels of organic matter: (a) low, (b) medium and (c) high. Points indicate the response of each sample (flufenacet in blue; pendimethalin in red) and the fitted model is shown by a solid black line (fitting separate lines to each soil type significantly improved the fit of the model but there was no significant difference between the two herbicides, so a single line was fitted across both). Dose is given as a proportion of recommended field rate.

A Cedergreen–Ritz–Streibig model (type C) best described the survival data. The fit of the model was significantly improved by incorporating soil organic matter but there was no improvement in the model when two separate curves were fitted for each active ingredient. In the model incorporating soil organic matter, parameters *b*, *d* and *γ* could be fixed to be common for all soil types. However, fixing the *η* parameter caused a significant change in the model and so this was allowed to depend on soil organic matter (Table 3). There is not a direct biological correspondence for the *η* parameter in the Cedergreen–Ritz–Streibig model but it does provide a lower bound on the ED50 level and so relates to the placement of the curve on the dose axis. This type of model accounts for hormesis: there were more survivors at low doses of herbicide than in the controls that received no herbicide (Figure 1).

**Table 3:** Fitted parameter values for the Cedergreen–Ritz–Streibig model used to describe the dose–response of the proportion of A. myosuroides seedlings surviving 6 weeks after the application of two pre‐emergence herbicides on three levels of soil organic matter. Common parameters were fitted for both herbicides

Parameter	Estimate	Standard error
*b*	1.777	0.2258
*d*	0.773	0.0451
*η* – low organic matter	0.097	0.0170
*η* – medium organic matter	0.234	0.0500
*η* – high organic matter	0.374	0.0850
*γ*	1.187	0.5743

The relative growth stage, as indicated by the number of tillers, at 6 weeks after spraying showed a three‐parameter log‐logistic response to the dose of herbicide applied. The fit of this model was significantly improved by fitting separate curves to each soil organic matter and herbicide and so their interaction was also assessed. Allowing an interaction between the two herbicides and soil organic matter also significantly improved the fit of the model. All parameters except *η* could be fixed to be common across treatments without significantly reducing the goodness of fit (Supporting Information Table S1). The plants grown in the lowest organic matter soil showed a reduction in the number of tillers at lower doses of herbicide than was observed for medium or high levels of soil organic matter. Flufenacet reduced the number of tillers by 50% at lower doses than was seen for pendimethalin (Supporting Information, Table S1; *η* represents ED50 value) but this difference was less marked on high organic matter soil (Supporting Information, Figure S1).

**Figure 2:**
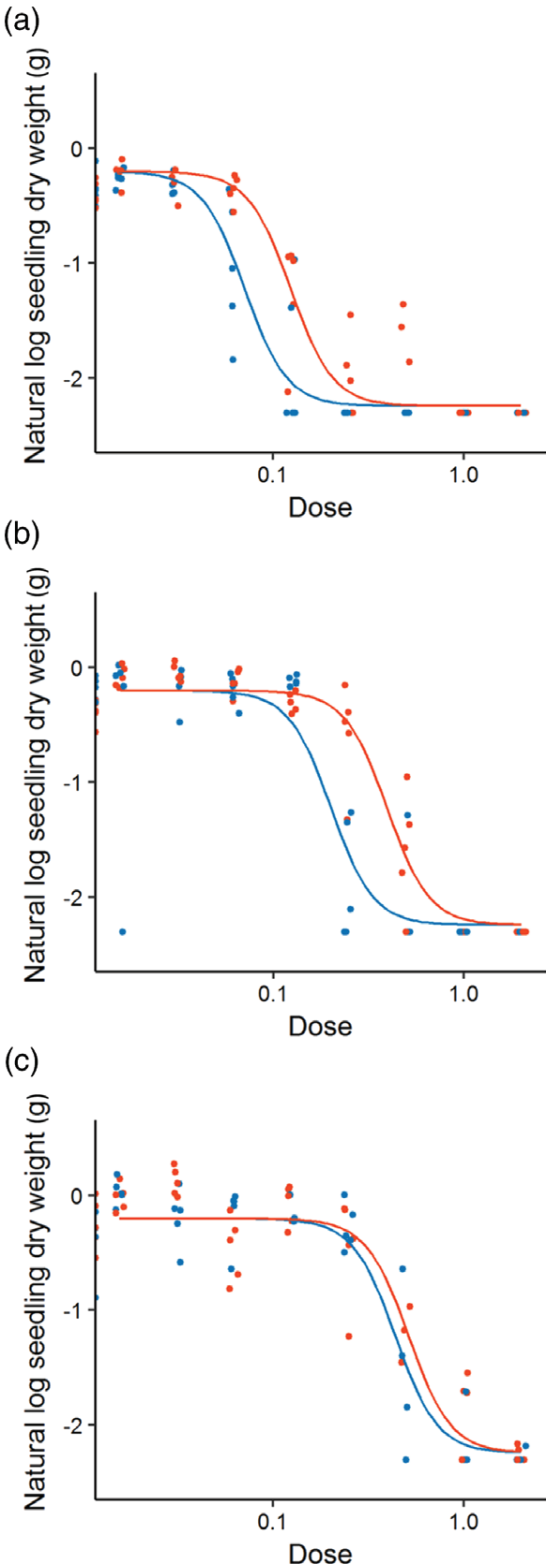
The log dry weight of seedlings surviving 6 weeks after the application of two pre‐emergence herbicides on soil with varying levels of organic matter: (a) low, (b) medium and (c) high. Points indicate the response of each sample and the fitted model is shown by a solid line (flufenacet in blue; pendimethalin in red). Dose is given as a proportion of recommended field rate.

The size of the seedlings as indicated by dry biomass measurements (natural logarithms) showed a four‐parameter log‐logistic response to dose. Allowing separate curves to be fitted for each soil type and each herbicide significantly improved the model (*P* < 0.05) so we tested for an interaction between them. This again significantly improved the fit of the model. The *b*, *c* and *d* parameters of the logistic curve could be fixed to be common across all soil types and both herbicides, leaving only the *η* parameter to vary (Table 4). For the logistic curve, the *η* parameter signifies the ED50 value indicating that the positioning of the curves on the dose axis was affected by soil type and the herbicide applied. For both herbicides, the ED50 was lowest on low organic matter soil and increased with organic matter. For pendimethalin, the ED50 was higher than it was for flufenacet, across all soil types. However, this difference was less marked on the highest organic matter soil (Figure 2).

**Table 4:** Fitted parameter values for the log‐logistic model used to describe the dose–response of the log dry biomass of A. myosuroides seedlings surviving 6 weeks after the application of two pre‐emergence herbicides on three levels of soil organic matter

Parameter	Estimate	Standard error
*b*	3.895	0.5442
*c*	−2.242	0.0402
*d*	−0.204	0.0287
*η* – low organic matter, flufenacet	0.071	0.0056
*η* – low organic matter, pendimethalin	0.124	0.0088
*η* – medium organic matter, flufenacet	0.200	0.0125
*η* – medium organic matter, pendimethalin	0.393	0.0315
*η* – high organic matter, flufenacet	0.437	0.0319
*η* – high organic matter, pendimethalin	0.514	0.0487

Despite significant effects of the herbicide used, the dose applied, and the amount of soil organic matter on plant numbers, growth stage, and size at 6 weeks, these effects were diminished as the plants were grown on to maturity. There were few survivors at the higher doses used, particularly on the low organic matter treatment, and so there was less replication in this part of the experiment looking at sublethal effects. There was no significant change in the Julian day of flowering (*P* < 0.05) with herbicide or dose, nor a response to soil organic matter.

The number of seed heads and the dry weight of seed produced by each plant were conserved across the full range of doses and there was no significant effect of dose or herbicide in any model. However, a significant response to soil organic matter was detected (*P* < 0.001; ANOVA) with the number of heads per plant increasing with organic matter (Table 5). It is likely that this is a result of a ‘fertilising’ effect of the additional organic matter on weed growth.

**Table 5:** The number of seed heads and the dry weight of that seed for plants subjected to all doses of flufenacet and pendimethalin at the pre‐emergence stage on soil with varying levels of organic matter

Soil organic matter	Number of seed heads		Dry weight of seeds (g)	
	Mean	SEM	Mean	SEM
Low	61.38	4.343	4.637	0.357
Medium	106.7	8.051	8.251	0.587
High	145.2	8.126	12.330	0.811

SEM, standard error of the mean.

**Figure 3:**
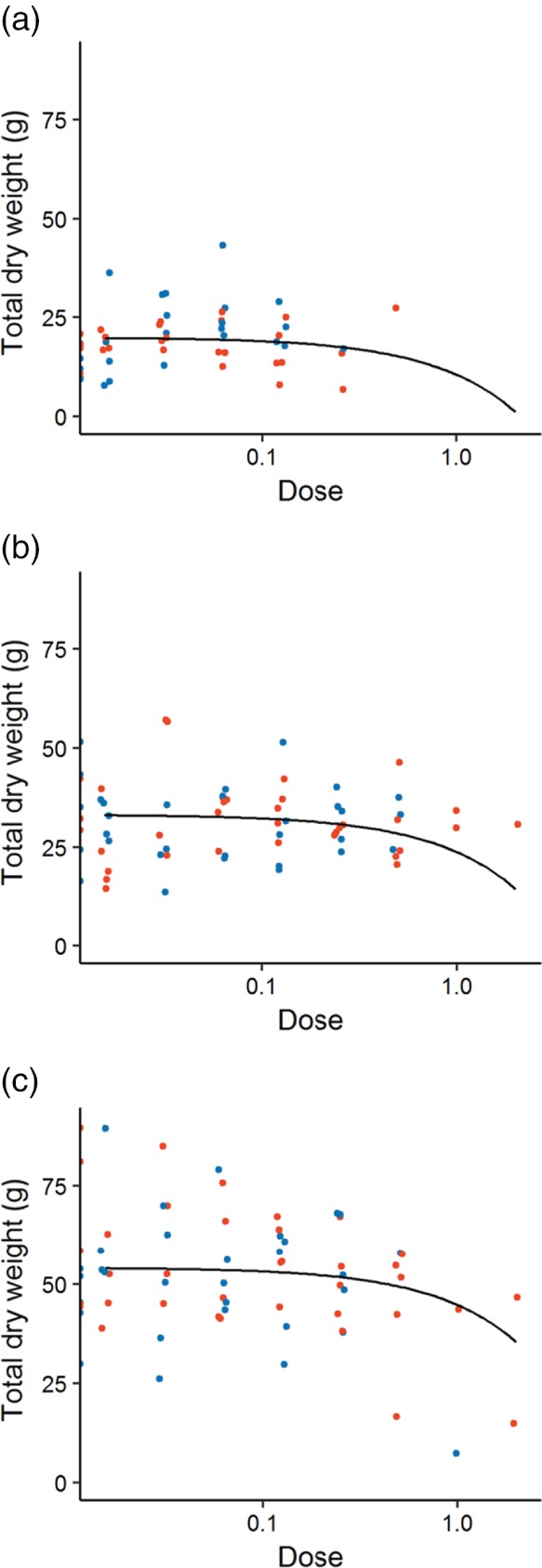
The total dry weight of mature plants after the application of two pre‐emergence herbicides on soil with varying levels of organic matter: (a) low, (b) medium and (c) high. Points indicate the response of each sample (flufenacet in blue; pendimethalin in red) and the fitted model is shown by a solid black line. (The fit of the model was significantly improved by allowing separate lines to be fitted to each soil type, yet there was no significant difference between the two active ingredients, so a single line was fitted across both herbicides.) Dose is given as a proportion of recommended field rate.

The total dry weight of the mature plants was, however, affected by dose as well as soil organic matter and was best described by a linear model with parallel lines and different intercepts for each soil type (Figure 3). The greater the herbicide dose at the pre‐emergence stage, the lower the total plant biomass at maturity, irrespective of the active herbicidal ingredient. The soil on which a plant was grown, however, caused a difference in the overall size of those plants, with the largest plants growing on high organic matter soil (Figure 3). The fact that there was a dose–response for total biomass but not for seed production indicates variability in partitioning of assimilate between the treatments.

Seed viability was not affected by the active ingredient or dose received by the parent plant. However, a significant response to soil organic matter was detected (P < 0.001; ANOVA), with plants grown on low organic matter soil producing seed with a lower percentage of seeds germinating (44.7 ± 0.14%) than those on medium (52.4 ± 1.18%) or high organic matter soil (54.8 ± 1.04%).

### 3.1 Modelling

To predict the seed production under each treatment scenario included in our experiments (three levels of soil organic matter and two herbicides at nine doses), we used the predictions from the statistical models fitted to the survival data and the size of the seedlings at 6 weeks as inputs into a crop–weed competition model. As survival was not significantly affected by the choice of herbicide, we adjusted survival by organic matter and dose. However, the log‐logistic model describing the dose–response of seedling biomass did significantly differ according to the herbicide used and so we were able to use different inputs for flufenacet and pendimethalin here. In each case, the seedling size at zero dose on each soil was used as a baseline for that soil and adjustments were made in terms of percentage size reduction. We were therefore able to simulate all three soils, both herbicides and the full range of doses, both with and without competition. The survival rates and the size reduction of individuals compared with the unsprayed plants are shown in Table 6.

**Table 6:** The predicted proportion of seedlings surviving and the dry weight of those seedlings (as a proportion of the predicted dry weight for those that received no herbicide on the same soil type) surviving 6 weeks after the application of either flufenacet or pendimethalin on soil with varying levels of organic matter. Predictions for seedling survival come from a Cedergren–Ritz–Streibig model fitted to experimental data. Fitting separate lines to each soil type significantly improved the fit of the model but there was no significant difference between the two herbicides, so a single line was fitted across both. Predictions for seedling dry weight come from a four‐parameter log‐logistic model fitted to experimental data. Fitting separate lines to each soil type and to each herbicide significantly improved the fit of the model, so separate lines were fitted to each soil × herbicide combination. Dose is given as a proportion of recommended field rate

Herbicide dose	Low organic matter		Medium organic matter		High organic matter	
	Survival	Dry weight	Survival	Dry weight	Survival	Dry weight
Flufenacet:						
0	0.773	1.000	0.773	1.000	0.773	1.000
1/64	0.812	0.994	0.837	1.000	0.841	1.000
1/32	0.780	0.924	0.860	0.999	0.873	1.000
1/16	0.642	0.464	0.853	0.978	0.897	0.999
1/8	0.389	0.160	0.749	0.754	0.870	0.985
¼	0.168	0.132	0.500	0.237	0.714	0.813
½	0.059	0.130	0.234	0.138	0.425	0.278
1	0.019	0.130	0.085	0.131	0.180	0.141
2	0.006	0.130	0.028	0.130	0.062	0.131
Pendimethalin:						
0	0.773	1.000	0.773	1.000	0.773	1.000
1/64	0.812	0.999	0.837	1.000	0.841	1.000
1/32	0.780	0.991	0.860	1.000	0.873	1.000
1/16	0.642	0.876	0.853	0.998	0.897	0.999
1/8	0.389	0.354	0.749	0.977	0.870	0.992
¼	0.168	0.147	0.500	0.742	0.714	0.890
½	0.059	0.131	0.234	0.231	0.425	0.381
1	0.019	0.130	0.085	0.137	0.180	0.150
2	0.006	0.130	0.028	0.131	0.062	0.132

In each case, the multiple simulations for each year generated a classic hyperbolic response curve when seed production was plotted against weed density. When crop competition was excluded, there were large levels of seed production across all treatments and we saw a clearly defined asymptote in seed production. Where crop competition was included in the model, weed densities were much lower and so we only observed the initial phase of this response curve and there was no asymptote in weed seed production (Supporting Information Figure S2).

There were large inter‐annual differences in the balance between crop and weed competition, which reflects the behaviour of the system in the field.^22^ For both the absence and presence of competition, a Cedergreen–Ritz–Streibig model (type C) best described the predicted weed seed production when plotted against herbicide dose. This reflects the type of model that was used for the input survival data—showing the importance of herbicide survival in the resultant seed return. The fit of the model was significantly improved in both cases (with and without crop competition) by allowing it to vary with both soil type and herbicide. This shows that sublethal effects of a reduced seedling size are important in determining seed return as mortality was fixed across both herbicides and so the differences seen here are only to do with sublethal effects.

**Figure 4:**
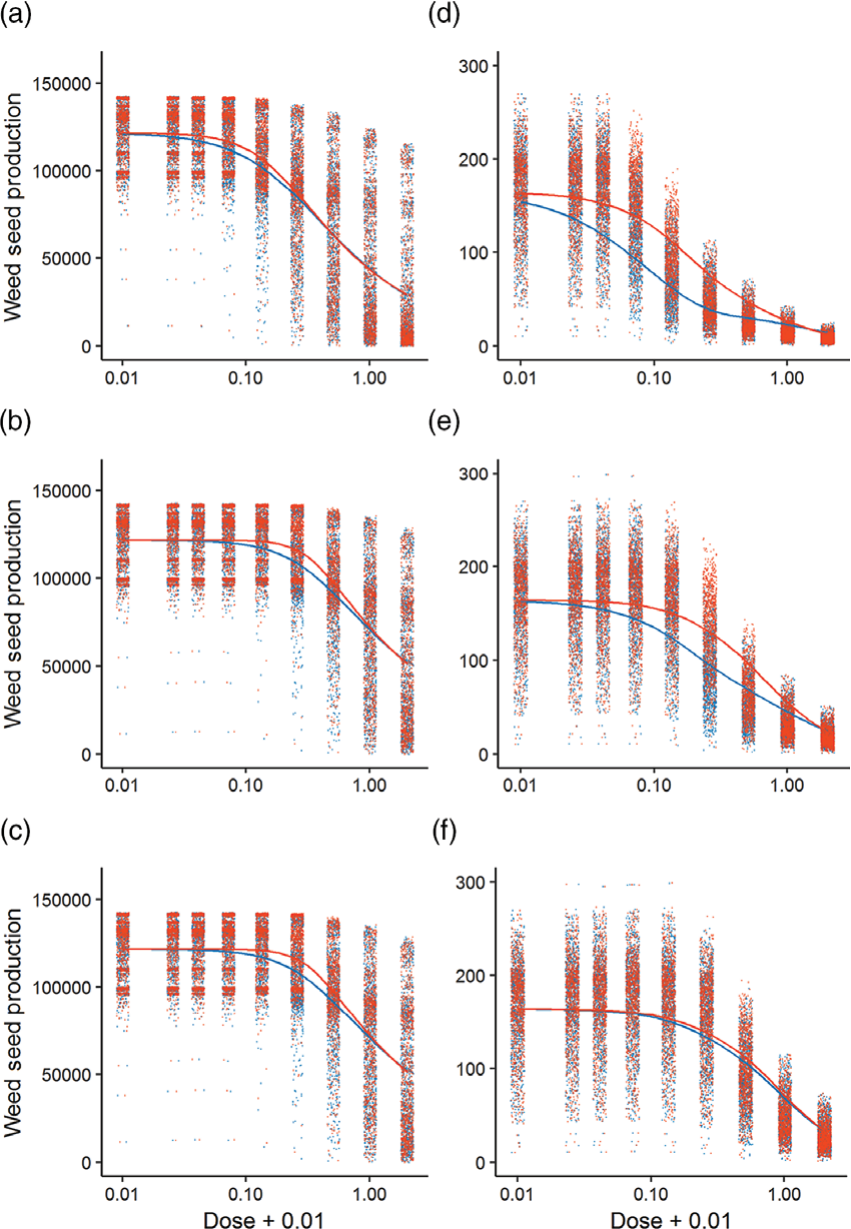
Weed seed production in the absence (a–c) and presence (d–f) of crop competition: outputs from 100 simulations for each of 10 years of weather data from the INTERCOM model across a full range of doses of herbicide application from 0 to 2× field rate on soil with varying amounts of soil organic matter: (a, d) low, (b, e) medium and (c, f) high. Points indicate the response of each model simulation and the fitted model is shown by a solid line (flufenacet in blue; pendimethalin in red). Dose is given as a proportion of recommended field rate. Seed production is on a square root scale where crop competition is included (d–f).

In the model output, we observe a reduction in seed return at high doses in the absence of competition (Figure 4a‐c). However, the spread of the predictions from the model becomes particularly wide as the dose increases. At low doses, seed production reaches an asymptote; this is particularly clear on soil with high organic matter as it is only once doses reach ½× field rate that we begin to see any reduction in seed return in the absence of competition (Figure 4c).

When we include crop competition in the simulation model (Figure 4d‐f), seed production is generally much lower and we see more of a decline across a wider range of doses. It is on low organic matter soil that we observe the lowest seed production, with flufenacet providing the greatest level of control. As we increase the organic matter, seed production increases and the difference between the two herbicides becomes less. When we consider the seed production at a field rate dose of herbicide, we can see that, whilst on low and medium organic matter soil it is fairly close to its lower asymptote, on high organic matter soil there is still a high level of seed production.

The difference in ED50s between the pot experiment and predicted seed return (Table 7) can be considered to be indicative of the capacity of *A. myosuroides* to compensate for the combined lethal and sublethal effects of the herbicides. The ED50 was higher for seed production than survival or seedling dry weight, emphasising the importance of managing a suppressive crop canopy to support herbicide use. The lower sublethal effects observed on soil with high organic matter were reflected in a disproportionally greater capacity of the weeds to compensate for herbicide activity, reflecting asymmetric crop–weed competition as size differences at canopy closure are magnified through the season.

**Table 7:** The predicted ED50 for seedling survival, seedling biomass and weed seed production. For seedling survival, values are obtained from the Cedergreen–Ritz–Streibig dose–response curve fitted to the experimental data (fitting separate lines to each soil type significantly improved the fit of the model but there was no significant difference between the two herbicides so a single line was fitted across both). For seedling biomass, values are obtained from the log‐logistic dose–response curve fitted to the experimental data (separate lines were fitted for each soil type and each herbicide). For weed seed production, values are obtained for the Cedergreen–Ritz–Streibig dose–response curve fitted to the output from the crop competition model (separate curves were fitted to each herbicide and each soil type)

Herbicide	Soil organic matter	Seedling survival		Seedling biomass		Weed seed production	
		ED50	SE	ED50	SE	ED50	SE
Flufenacet	Low	0.1258	0.022	0.0711	0.006	0.1226	0.002
	Medium	0.3279	0.055	0.1997	0.013	0.3330	0.004
	High	0.5466	0.088	0.4374	0.032	0.6168	0.007
Pendimethalin	Low	0.1259	0.022	0.1238	0.009	0.1577	0.002
	Medium	0.3279	0.055	0.3932	0.032	0.4465	0.005
	High	0.5466	0.088	0.5137	0.049	0.6611	0.007

SE, Standard error.

These results, in combination, show us that soil with greater organic matter content generally leads to poorer control with flufenacet and pendimethalin than can be achieved on soil with less organic matter. For traits that are affected by the choice of herbicide, namely those relating to early growth, there is a greater level of efficacy achieved by flufenacet than pendimethalin, particularly on soil with lower organic matter. In the absence of competition, survivors can recover to produce large amounts of seed, in some cases (as we observed in our experiment) the same amount of seed as if no herbicide were applied. However, the simulation model showed that the reduction in size of seedlings following the application of pre‐emergence herbicides leads to increased competition by the crop. Whilst this indicates that in the presence of competition the lack of sublethal effects observed in our experiments does not hold, it does indicate that on high organic matter soil where *A. myosuroides* seedlings can survive field‐rate doses of pre‐emergence herbicide it is possible for them to recover and produce non‐negligible amounts of seed.

## 4 DISCUSSION

Our results show that soil organic matter plays an important role in the control of *A. myosuroides* achieved by flufenacet and pendimethalin. The artificial soils used in the pot experiments reflected typical ranges of soil organic matter in UK arable fields and, although the results cannot be extrapolated directly to the field, the range of efficacies generated for the model simulations represented a realistic range for assessing the implications of variable pre‐emergence herbicide activity for weed seed production.

As far as seedling survival is concerned, the placement of the curve on the dose axis was altered significantly depending on the levels of organic matter in the soil. Soil with a greater concentration of organic matter shifts the dose–response curve to the right, meaning a higher dose of herbicide is required to achieve the same reduction in survival. Similarly, the size of the plants after 6 weeks was also strongly affected by soil organic matter, with surviving plants grown in soil with much organic matter typically being larger than those grown in soil with less organic matter. This would indicate that on higher organic matter soil, where pre‐emergence herbicides are used for *A. myosuroides* control, there are likely to be more survivors than on lower organic matter soil and those surviving individuals will be larger and so more likely to be able to compete well with the crop plants. It is also likely that soil with high organic matter is better suited for *A. myosuroides* growth and competition as it has a higher capacity for moisture retention.^25^ We would, therefore, expect an additive effect leading to enhanced weed fitness on these soils. Further development of the model to simulate these effects of soil heterogeneity on weed growth (and also on the relationship between mature biomass and seed production) would give additional insight into the final impact of variable herbicide efficacy on weed seed production. However, we hypothesise that incorporating these processes will only further decrease the fitness penalty of sublethal herbicide effects for weeds growing on soils with relatively high organic matter, further emphasising the need to consider enhanced weed management on these areas of the field.

The size benefit conferred by high organic matter soil appears to hold true throughout the plants' life‐cycle, with plants grown in soil with higher organic matter reaching greater mature biomasses and producing more seed in the absence of competition. In addition to this, there were few sublethal effects of the herbicide dose, amongst the variables we measured here in the absence of competition. Whilst soil organic matter still plays an important role, we observe no cost in terms of seed production and only a small cost in terms of total biomass production to having received a higher dose of either pre‐emergence herbicide. The seed produced by plants that received high doses of either pre‐emergence herbicide also show similar viability to unsprayed plants, implying that plants adjust partitioning of resources such that fecundity is not compromised. In a crop‐free environment, this has no cost but would reduce the plant's competitive ability when growing with a crop.

It is possible that sublethal effects of high doses of herbicides could be masked in our experiments as a consequence of the use of pots rather than field studies. However, the magnitude of differences observed between the soil organic matter levels indicates that it is unlikely, at least in the low organic matter treatment, that the pots were limiting. However, further field trials could confirm this. Our modelling study shows that when competing with a crop the reduction in biomass at the seedling stage could have severe consequences for *A. myosuroides* seed production, yet even for field‐rate application of herbicide seed production is non‐negligible, with most seed return on high organic matter soil. Our results also indicate that this reduction in seed production across different soil types is not only attributable to increased competition with the crop on the different soil types but also varies with herbicide choice as sublethal effects come into play.

The work we present here supports the claims of others^8^, ^11^ that pre‐emergence herbicidal control is affected by soil organic matter, even within the small range of organic matters typical of the UK arable landscape. Despite this, the label recommendation for many of these herbicides suggests that they remain effective up to 10% organic matter. This may have strong implications for minimal‐ and no‐tillage systems where there is an increase in the amount of organic matter in the topsoil as this could mean decreased levels of control by pre‐emergence herbicides.

We have established here that the control of *A. myosuroides* by pre‐emergence herbicides can be impacted strongly by soil organic matter. This highlights an opportunity for further research into whether these results hold true in a field situation. It also raises questions about the efficacy of other active ingredients applied to the soil as well as mixtures of multiple active ingredients across a range of soil properties.

In terms of impacts on *A. myosuroides* management, herbicide application by soil type is possible as many farmers have soil maps of their farms and the uptake of precision agriculture is advancing. So, in fields where there are within‐field gradients of organic matter, it should be possible to adjust the application rate of the post‐emergence herbicide to account for this. However, it may be that further work is required to determine active ingredients or mixtures thereof that are less impacted by soil organic matter and perhaps tailor herbicide programmes to the soil properties within fields. We have also demonstrated the importance of crop competition in supporting pre‐emergence herbicides in the context of a variable soil environment. This effect could be further enhanced (so reducing the capacity of weeds to compensate for sublethal herbicide effects) through cultural control options such as increased seed rate and the use of competitive cultivars.^26^ The effective combination of integrated weed management and precision weed management, therefore, cannot be achieved by studying chemical and agronomic weed control options in isolation but will require an assessment of their combined impacts across the whole growing season of the type presented in this study.

Our results support our first hypothesis that increasing soil organic matter would lead to decreased efficacy of both flufenacet and pendimethalin in the control of *A. myosuroides*. We expect that this is attributable to adsorption of herbicide.^7^ The differences between the two herbicides could be attributable to different levels of adsorption, as was described by Nordmeyer^8^ for pendimethalin and chlortoluron. Our second hypothesis was that sublethal doses would lead to fitness costs causing reduced growth and fecundity. We observed very little evidence of this in the experiments, with only a small effect of dose on total biomass but no observable effect on seed return. However, the reduction in size at 6 weeks following spraying, when input into a crop competition model, suggests that fitness costs play an important role in reducing seed return with plants sprayed with flufenacet, which led to reduced biomass at 6 weeks, producing less seed overall.

## Supporting information


**Table S1.** Fitted parameter values for the log‐logistic model used to describe the dose–response of the number of tillers per plant of A. myosuroides seedlings surviving 6 weeks after the application of two pre‐emergence herbicides on three levels of soil organic matter
**Figure S1.** The number of tillers per plant surviving 6 weeks after the application of two pre‐emergence herbicides on soil with varying levels of organic matter: (a) low, (b) medium and (c) high. Points indicate the response of each sample and the fitted model is shown by a solid line (flufenacet in blue; pendimethalin in red). Dose is given as a proportion of recommended field rate.
**Figure S2.** Outputs from 100 simulations for each of 10 years of weather data from the INTERCOM model (a) in the absence of crop competition and (b) in the presence of crop competition. Data points when there is no size penalty to having been sprayed with pre‐emergence herbicide are shown in green (mortality adjusted according to inputs in Table 6; no reduction in seedling biomass), and those with a size penalty for a sublethal dose of herbicide are shown in purple. (Mortality and seedling biomass adjusted according to inputs in Table 6.) A linear model that best describes the data is shown with 95% confidence intervals. [For panel (a), the linear model was fitted to log densities. The back transformed model is shown here.]
