# Supplementary material for: The implications of spatially variable pre‐emergence herbicide efficacy for weed management
Source: Pest Manag Sci. 2017 Dec 14;74(3):755–65. doi: 10.1002/ps.4784 (PMC5814844; doi:10.1002/ps.4784)
Supplement: Supplementary file 1 — Table S1. Fitted parameter values for the log‐logistic model used to describe the dose–response of the number of tillers per plant of A. myosuroides seedlings surviving 6 weeks after the application of two pre‐emergence herbicides on three levels of soil organic matter Figure S1. The number of tillers per plant surviving 6 weeks after the application of two pre‐emergence herbicides on soil with varying levels of organic matter: (a) low, (b) medium and (c) high. Points indicate the response of each sample and the fitted model is shown by a solid line (flufenacet in blue; pendimethalin in red). Dose is given as a proportion of recommended field rate. Figure S2. Outputs from 100 simulations for each of 10 years of weather data from the INTERCOM model (a) in the absence of crop competition and (b) in the presence of crop competition. Data points when there is no size penalty to having been sprayed with pre‐emergence herbicide are shown in green (mortality adjusted according to inputs in Table 6; no reduction in seedling biomass), and those with a size penalty for a sublethal dose of herbicide are shown in purple. (Mortality and seedling biomass adjusted according to inputs in Table 6.) A linear model that best describes the data is shown with 95% confidence intervals. [For panel (a), the linear model was fitted to log densities. The back transformed model is shown here.] [file PS-74-755-s001.docx]

**Table S1.** Fitted parameter values for the log-logistic model used to describe the dose–response of the number of tillers per plant of A. myosuroides seedlings surviving 6 weeks after the application of two pre-emergence herbicides on three levels of soil organic matter

| Parameter | Estimate | Standard error |
| --- | --- | --- |
| $b$ | 2.870 | 0.3570 |
| $d$ | 4.101 | 0.0698 |
| $\eta$ – low organic matter, flufenacet | 0.086 | 0.0083 |
| $\eta$ – low organic matter, pendimethalin | 0.154 | 0.0145 |
| $\eta$ – medium organic matter, flufenacet | 0.276 | 0.0264 |
| $\eta$ – medium organic matter, pendimethalin | 0.409 | 0.0423 |
| $\eta$ – high organic matter, flufenacet | 0.395 | 0.0330 |
| $\eta$ – high organic matter, pendimethalin | 0.516 | 0.0083 |

| a) Low organic matter | 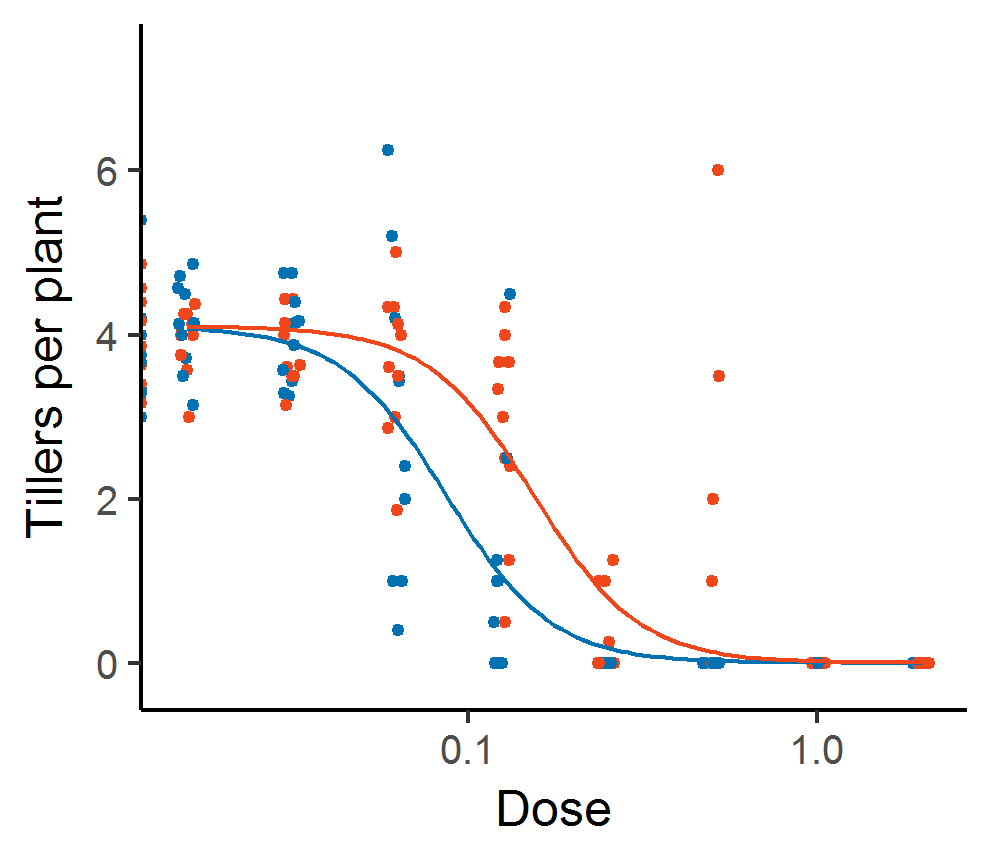 |
| --- | --- |
| b) Medium organic matter | 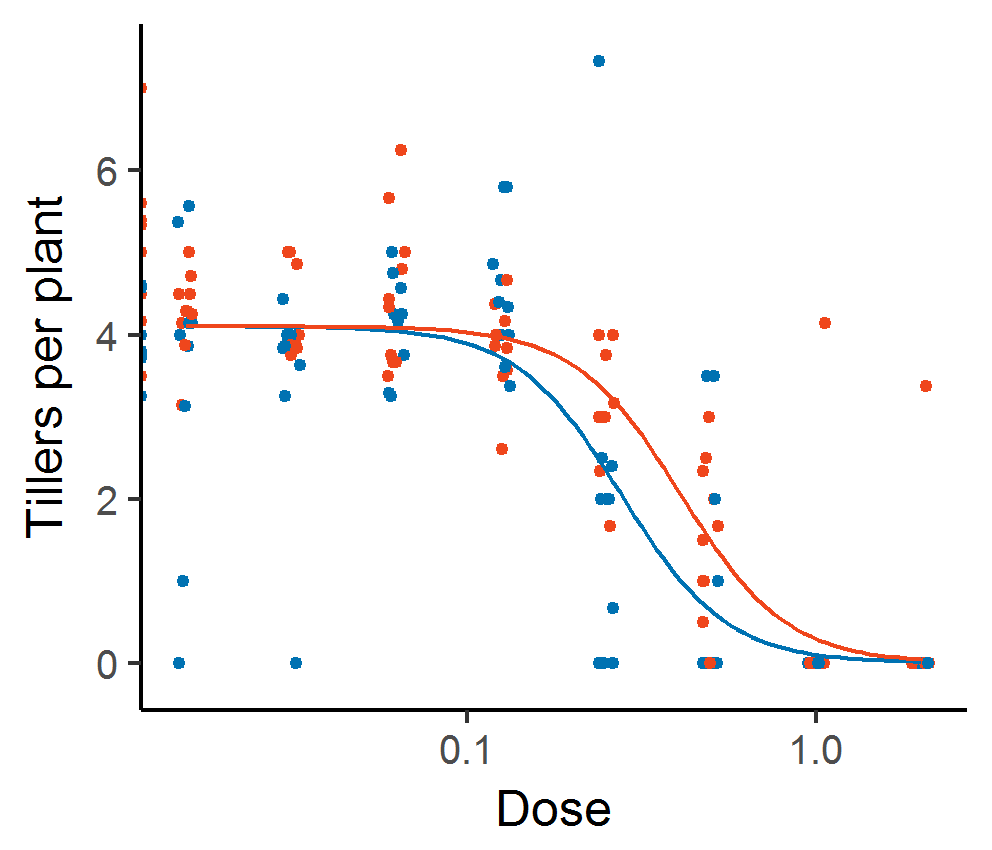 |
| c) High organic matter | 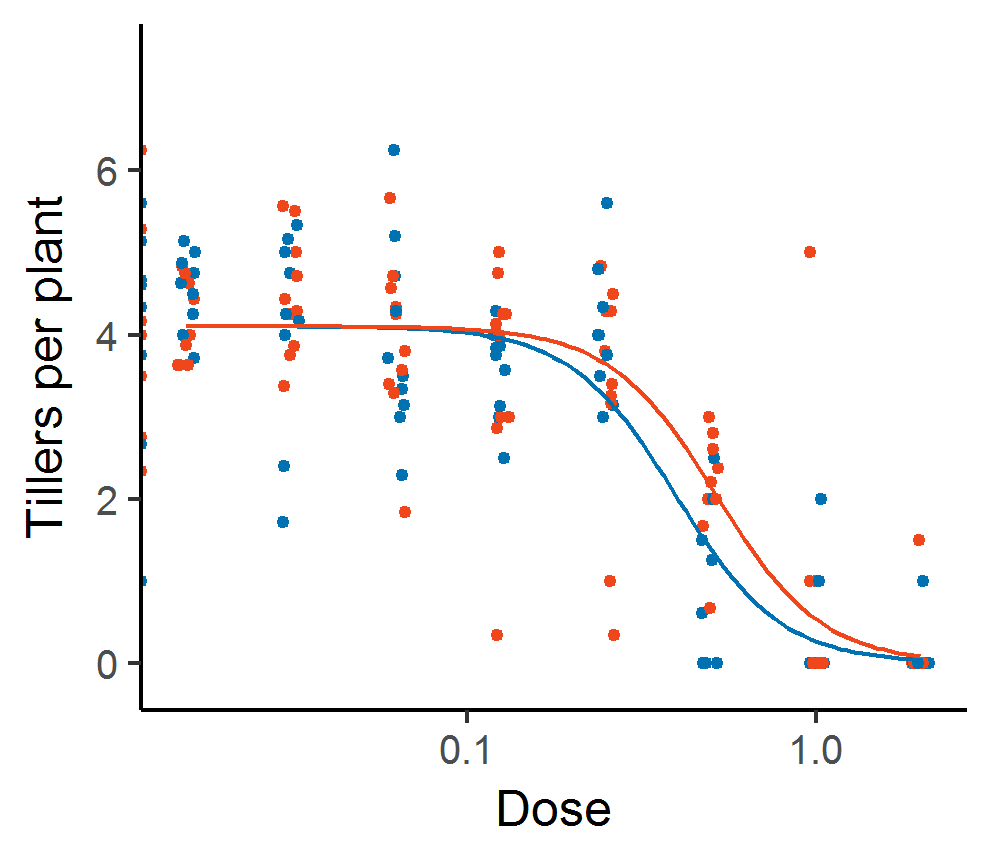 |

**Figure S1.** The number of tillers per plant surviving 6 weeks after the application of two pre-emergence herbicides on soil with varying levels of organic matter: (a) low, (b) medium and (c) high. Points indicate the response of each sample and the fitted model is shown by a solid line (flufenacet in blue; pendimethalin in red). Dose is given as a proportion of recommended field rate.

| 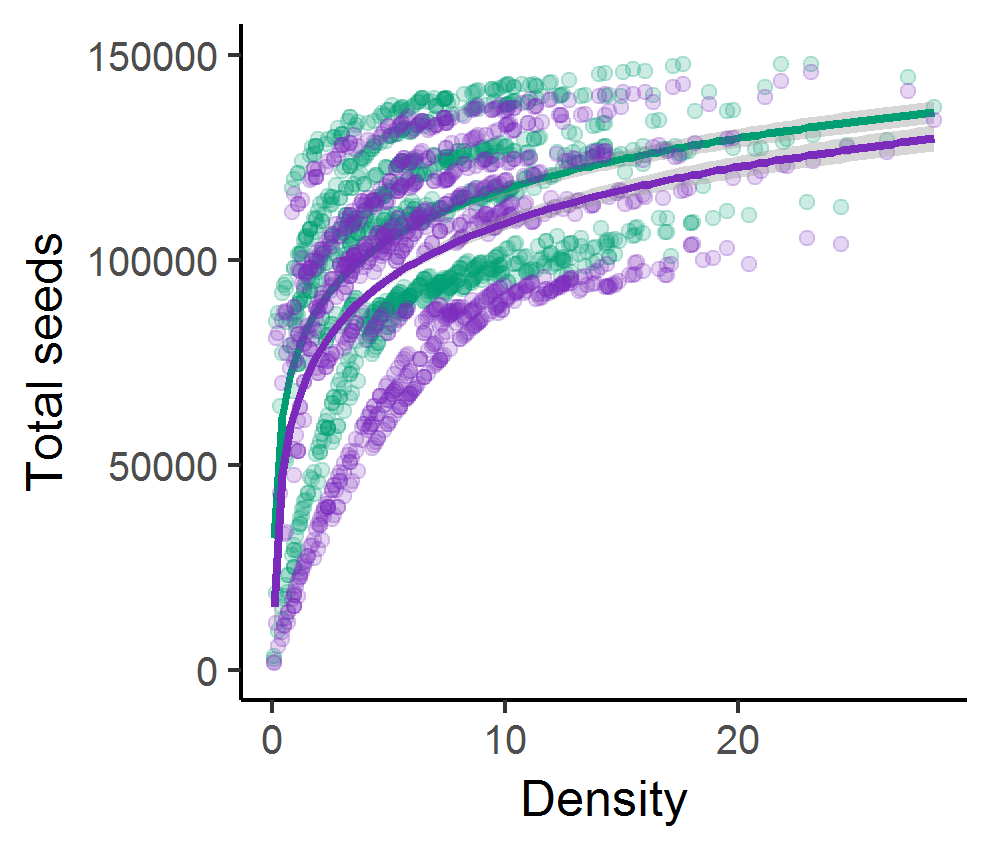 | 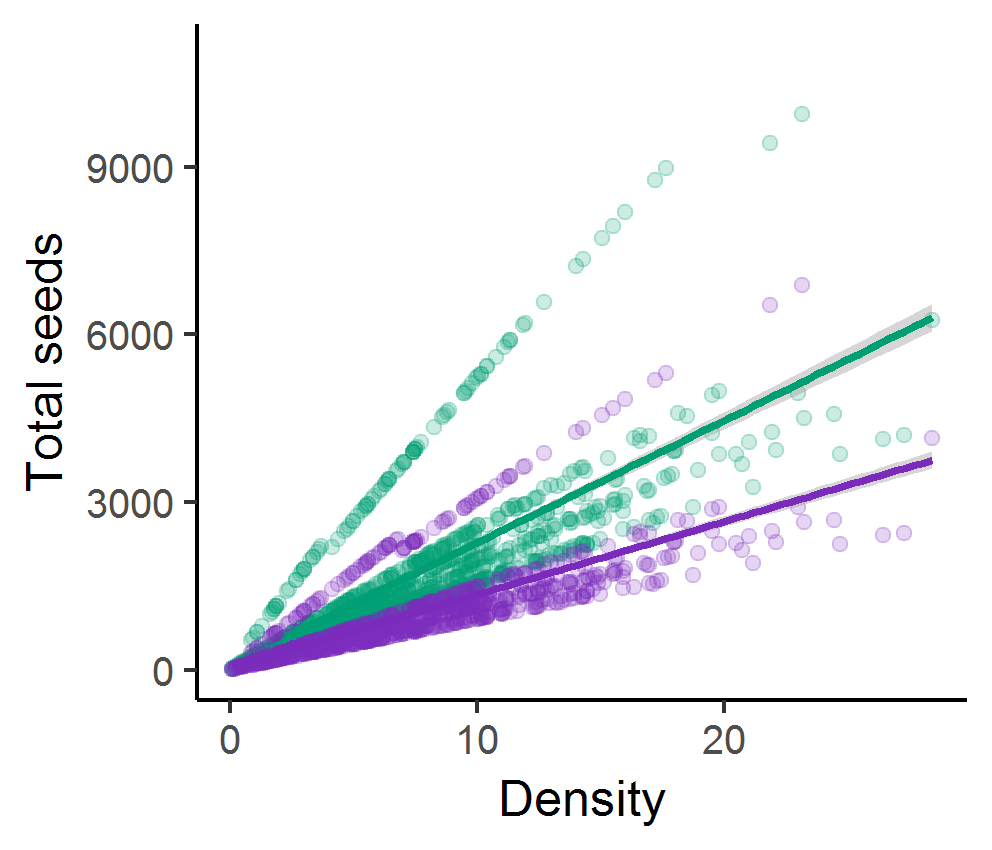 |
| --- | --- |

**Figure S2.** Outputs from 100 simulations for each of 10 years of weather data from the INTERCOM model (a) in the absence of crop competition and (b) in the presence of crop competition. Data points when there is no size penalty to having been sprayed with pre-emergence herbicide are shown in green (mortality adjusted according to inputs in Table 6; no reduction in seedling biomass), and those with a size penalty for a sublethal dose of herbicide are shown in purple. (Mortality and seedling biomass adjusted according to inputs in Table 6.) A linear model that best describes the data is shown with 95% confidence intervals. [For panel (a), the linear model was fitted to log densities. The back transformed model is shown here.]
